# Diagnosis and therapy of acute urolithiasis caused by melamine contamination in infant formula milk

**DOI:** 10.3892/etm.2013.1011

**Published:** 2013-03-15

**Authors:** JUN HE, WEI ZHENG, YAOWANG ZHAO, LI LIU, JIANG YONG

**Affiliations:** Department of Urology, Hunan Children’s Hospital, Changsha, Hunan 410007, P.R. China

**Keywords:** acute urolithiasis, melamine, infant formula milk

## Abstract

The aim of this study was to determine the clinical characteristics and diagnosis of severe, acute urolithiasis in infants and toddlers, caused by melamine contamination in infant formula milk. The clinical data of 28 patients were collected and analyzed. Among the 28 patients, 17 patients received indwelling ureteral stents by cystoscopy (60.71%) and 5 patients received open surgery to extract calculi (17.86%). Four patients received catheterization or diuretic, anti-inflammatory or antispasmodic treatment (14.28%). Two patients underwent a second open surgery to extract calculi (7.14%). Eventually, the stones were eliminated from 23 patients and 5 patients had residual stones. In the 5 patients with residual stones, 3 patients had kidney stones, hydronephrosis or unilateral ureteral stones, resulting in urinary obstruction following surgery. Urolithiasis in infants and toddlers caused by melamine contamination was diagnosed, with common symptoms, including acute anuria, oliguria and dysurias. Ultrasonic inspection may be used to successfully examine urinary stones. Computed tomography (CT) scanning further detects the position of the stones and the degree of edema. Ureteral stenting via cystoscopy is a useful method, resulting in minimal trauma and a positive prognosis.

## Introduction

The incidence of pediatric urolithiasis is ∼1–4%, with ∼40–50% of cases aged <5 years and >20% aged <2 years (1). It has been reported that there were 50,000 cases of pediatric urolithiasis, including 12,000 cases that were hospitalized and 4 cases that succumbed, due to melamine-contaminated milk in China in 2008 (2–7). These children were ∼1 year old with various clinical manifestations. Due to multiple calculi in the kidney, obstructive anuria, oliguria and dysuria appears, threatening the health of the patients. Understanding such severe acute cases of pediatric urolithiasis is of great importance for diagnosis and treatment.

Metabolic diseases, chronic infection and urinary tract malformations are the major cause of pediatric urolithasis, particularly with metabolic disease and chronic infection (8). However, a large-scale pediatric urolithasis outbreak in 2008 resulted from the ingestion of melamine-contaminated infant formula milk. Melamine is a pure white monoclinic crystalline substance. It is tasteless, slightly soluble in cold water and weakly alkaline (pKb 8). It hydrolyzes in strong acid or alkali solution. When cats were fed with increasing doses of melamine-cyanuric acid, the cats developed renal failure, with crystals in the kidneys (9,10). This was also reported in a rat study by Dobson *et al*(11). The melamine-cyanuric acid compound has low solubility, which leads to the formation of melamine cyanurate crystals in the kidney. It is considered that melamine-cyanuric acid is absorbed in the digestive tract and distributed to the whole body. It precipitates in the renal tubule for unknown reasons, which leads to progressive obstruction and degeneration (11).

Twenty-eight severe acute cases of pediatric urolithiasis were analyzed in the present study. The use of indwelling ureteral stent placement by cystoscopy, open surgery and conservative treatments, including catheterization and diuretic, anti-inflammatory and anti-spasmodic therapy, produced good effects.

## Materials and methods

### General information

Twenty-eight cases of severe pediatric urolithiasis, including 21 males and 7 females, were admitted to the Department of Urology, Hunan Children’s Hospital. The average age was 13.9 months, ranging from 4 months to 3 years, with 17 infants and 11 toddlers. All patients had received melamine-contaminated infant milk formula. Prior written informed consent was obtained from parents of each child and the study was approved by the ethics review board of the Hunan Children’s Hospital, Hunan, Changsha, China.

All patients had acute onset of anuria, oliguria or dysuria. There were another three patients who had multiple symptoms, including kidney and ureter stones, unilateral ureteral calculi and bladder calculi (Table I). They were all in critical condition at admission with renal dysfunction and electrolyte imbalance. Among them, 21 patients had anuria (75%), two patients had oliguria (7.14%) and two patients had dysuria (7.14%). A number of patients also had unexplained fever, diarrhea, recurrent nausea and vomiting. Upper urinary tract calculi mainly presented anuria, which accounted for 86.95%. Lower urinary tract calculi mainly presented oliguria and dysuria, which accounted for 80% (Table I). One child had heart failure, severe infection and renal failure and two patients had a coagulation function disorder, one of which was disseminated intravascular coagulation (DIC).

### Localization of urolithiasis

There were 23 cases of upper urinary tract calculi, of which five cases were simple ureteral stones, 15 cases were ureteral stones and kidney stones, one case was simple kidney stones and two cases were unilateral ureteral stones and hydronephrosis. There were three cases with upper urinary tract stones and bladder stones and two cases with urinary tract stones (one case with anterior urethral stones and one case with posterior urethral stones).

### Imaging examination

**Figure 1:**
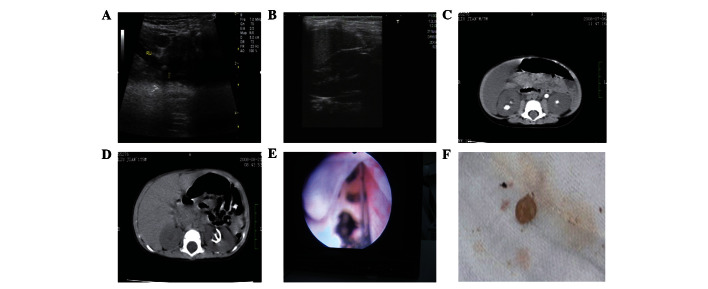
(A) Preoperative ultrasound. Stones (light dots, 10×6 mm) were observed in the uretero-pelvic junction. (B) Postoperative ultrasound. The stones had disappeared. No light dots were observed. (C) Preoperative computed tomography (CT). Multiple light dots were visible in the two kidneys. A strong light dot was observed in the left uretero-pelvic junction. (D) Postoperative CT. After indwelling epidural catheter placement, stones in the right kidney were discharged. In the left kidney, stones failed to be discharged. Therefore, open surgery was performed with the indwelling Double-J tube. (E) Cystoscopy indwelling ureteral stent. A deficit epidural catheter was inserted under a cystoscope. (F) Discharged stones. The stones were fragile and were easily crushed into a powder.

All cases had calculi, as detected by ultrasound. The majority of the stones affected the collecting system and ureters. Ureteral stones were mainly in the uretero-pelvic junction, the cross-iliac artery segment of the ureter and the ureter-bladder junction. Stones accumulated as powder, involving a large area. A light sound shadow was at the rear and a trailing edge was detectable, which is different from calcium oxalate stones. Stones obstructed the urinary tract (Figs. 1A and B). Computed tomography (CT) examination further clarified the location of the stones, hydronephrosis level and assessment of kidney function. There was mild to moderate hydronephrosis, bilateral ureteral expansion or no expansion, multiple kidney stones, multiple bilateral ureter with different locations or stones obstructing the junction of the renal pelvis and ureter, with no urine in the bladder (Figs. 1C and D).

### Treatment

The 28 children were admitted to hospital for examination of blood, liver, kidney and coagulation function, as well as ultrasound and CT scan. For children with upper urinary tract stones and anuria and oliguria, an indwelling ureteral stent was inserted with the aid of a cystoscope (Fig. 1E). For children with urinary tract stones, conservative treatment was applied. Stones in the urinary tract were pushed into the bladder. For children with bladder stones, conservative treatment or incision lithotripsy was administered (Table II). Postoperative examination was also performed.

### Statistical analysis

Data are expressed as mean ± standard error of the mean (SEM). The significance was analyzed by SPSS 16.0 statistical analysis software (SPSS Inc., Chicago, IL, USA) using one-way analysis of variance (ANOVA), with an inspection level of α=0.05.

## Results

### Outcomes of treatment

After the placement of a bilateral ureteral indwelling epidural catheter or Double-J tube by cystoscopy, urine was drained successfully in 17 cases (60.71%). Two days after surgery, renal function was detected to be normal. With infusion and antispasmodic treatments, stones were discharged in one week. Five patients received open-surgical lithotomy (17.86%). Four patients received catheterization and diuretic, anti-inflammatory and antispasmodic therapy (14.28%). Two patients received indwelling ureteral stent placement by cystoscopy and a second open surgery (7.14%). The discharged stones were gray, gray-yellow or white (Fig. 1F). The stones were fragile and were easily crushed into a powder.

### Renal function and electrolyte recovery

Renal function and electrolytes were abnormal in 22 children. However, following indwelling ureteral stent placement by cystoscopy, open surgery and conservative treatment, serum potassium levels in the majority of cases returned to normal within 1 day. Creatinine and urea nitrogen levels in the majority of cases returned to normal on the second day (Table III).

### Follow-up

When discharged from the hospital, pediatric urolithiasis was not detectable in 23 cases. In the five cases with residual stones, three cases had recurrence of urinary tract obstruction and two cases underwent re-examination by ultrasound one month after surgery, which identified kidney stones with hydronephrosis. All patients were followed up for two years.

## Discussion

In the 28 acute severe cases of pediatric urolithiasis in the present study, all patients had a history of contaminated milk and had no urinary tract abnormalities. The contaminated milk feeding time varied from 3 months to 1 year. The main complaint was anuria (75%) due to bilateral upper urinary tract calculi obstruction. Other complaints were oliguria and dysuria (14.28%). The onset of anuric renal dysfunction was acute and severe. Children were in a critical condition with hypertension, poor appetite and lack of energy, and internal environment disturbance, with or without mild edema. However, the anuria was temporary. Once the obstruction was relieved, urine was discharged. In this study, 21 children had anuria and they all discharged urine following indwelling ureteral stent placement by cystoscopy, open surgery and conservative treatment. Their renal dysfunction was prerenal and their kidney function recovered once the urinary tract obstruction was removed. There were 22 children with electrolyte disorders and their renal function and electrolyte disorders were all recovered one or two days after urine drainage. Nineteen of them underwent indwelling ureteral stent placement by cystoscopy and three underwent open surgery and received conservative treatment of urine alkalization following urine drainage.

The clinical diagnosis of infant urolithiasis caused by melamine relies on appropriate imaging examination. Ultrasound is the first option (12). Two-dimensional sonography provides location, size, shape, edge, number, echo and rear sound shadow, which indicates the hardness and degree of the loose structure, of the stones. It also shows the scope, degree of hydronephrosis, renal parenchymal compression and its internal structural changes. With the application of Doppler ultrasound (US), renal blood flow and urine flow in the renal hilum and ureter were observed in order to diagnose the obstruction. Clinically, US diagnosis of urolithiasis is extremely sensitive. A CT scan shows radioparentcalculus; however, it also aids the differential diagnosis. CT and US are the first options for urolithasis diagnosis. With US and CT scans, the locations of stones and anatomical deformity are observed for future treatment (13).

Non-open treatment is increasingly popular for pediatric urolithiasis, particularly extracorporeal shock wave lithotripsy (ESWL) and shock wave lithotripsy (SWL) (14,15). Moreover, with the development of cavity urinary technology, particularly fiber endoscopy, pneumatic lithotripsy is applied for treatment of pediatric urolithiasis (16). However, melamine-induced urolithiasis has its own characteristics: i) it easily causes bilateral urinary tract obstruction; ii) following indwelling ureteral stent placement by cystoscopy, urine discharges easily; and iii) the stones are loose and friable and when urine is drained, the stones self-discharge. In this study, indwelling ureteral stent placement by cystoscopy was applied. The amount of urine was recorded for 24 h and renal function and electrolytes were examined repeatedly. Antispasmodic anisodamine (654-2), urine alkalization, anti-infection and rehydration therapy were administered. When the vital signs were stable, the ureteral stents were removed. A CT scan was performed again to observe the amount of urine and to determine if the stones had been expelled. A volume of fluid and electrolytes was administered according to electrolyte values. Conservative therapy with a diuretic, antibiotics and rehydration was applied if the stones were small and the anuric time was short. If the obstruction remained, open surgery was performed. When the urine had been drained, the electrolyte levels of the children returned to normal one day after surgery and renal function recovered two days after surgery. It is essential that electrolyte balance disorders and infection are prevented in the polyuric period; therefore, children with preoperative concurrent heart failure, severe infection or coagulation disorders, including DIC, should be admitted to the intensive care unit (ICU) to get through the postoperative infection, polyuric and coagulation disorders or DIC periods.

In conclusion, pediatric urolithiasis caused by melamine forms quickly and is acute. It mainly presents anuria, oliguria and dysuria. CT and US are the first choice for diagnosis. Indwelling ureteral stent placement by cystoscope causes minimal injury and provides excellent results.

## Figures and Tables

**Table I:** Localization of stones and clinical manifestations.

Clinical manifestations	Kidney and ureter stones	Simple kidney stones	Unilateral ureteral calculi	Simple bilateral ureteral calculi	Bladder calculi	Urinary tract stones	Total
Anuria	13	1	1	5	1	0	21
Oliguria	1	0	0	0	1	0	2
Dysuria	0	0	0	0	0	2	2
Others	1	0	1	0	1	0	3
Total	15	1	2	5	3	2	28

**Table II:** Localization of stones and related treatment.

Related treatment	Kidney and ureter stones	Simple kidney stones	Unilateral ureteral calculi	Simple bilateral ureteral calculi	Bladder calculi	Urinary tract stones	Total
Cystoscopy	12	0	1	3	1	0	17
Open surgery	1	0	1	1	1	1	5
Cystoscopy + open surgery	1	0	0	1	0	0	2
Conservative treatment	1	1	0	0	1	1	4

**Table III:** Renal function recovery following treatment.

Time	Serum potassium (mM)	BUN (mM)	Cr (*μ*M)	CO_2_CP (mM)
Before treatment	5.22±0.25	18.86±1.95	390.2±27.7	18.74±0.68
1 day after treatment	4.38±0.23	12.00±1.43	185.2±23.3	19.66±0.93
2 days after treatment	3.79±0.16	7.00±1.13	107±17.1	20.02±0.58
P-value	<0.001	<0.001	<0.001	=0.46

BUN, blood urea nitrogen; Cr, creatinine; CO_2_CP, carbon dioxide combining power.
